# Behavioral Changes During Development of Chronic Kidney Disease in Rats

**DOI:** 10.3389/fmed.2019.00311

**Published:** 2020-01-09

**Authors:** Emese Renczés, Martin Marônek, Alexandra Gaál Kovalčíková, Diana Vavrincová-Yaghi, L'ubomíra Tóthová, Július Hodosy

**Affiliations:** ^1^Institute of Molecular Biomedicine, Faculty of Medicine, Comenius University in Bratislava, Bratislava, Slovakia; ^2^Department of Paediatrics, National Institute of Children's Diseases and Faculty of Medicine, Comenius University in Bratislava, Bratislava, Slovakia; ^3^Department of Pharmacology and Toxicology, Faculty of Pharmacy, Comenius University in Bratislava, Bratislava, Slovakia; ^4^Institute of Phsysiology, Faculty of Medicine, Comenius University in Bratislava, Bratislava, Slovakia

**Keywords:** cognition, dementia, mental health, neurological complications, renal failure

## Abstract

Decreased renal function due to chronic kidney disease (CKD) is associated with anxiety and cognitive decline. Although these mental disorders are often obvious in late stage renal disease patients, they might be unnoticeable or are neglected in early stages of the CKD development. Associations between renal and cognitive dysfunction have been indicated by studies performed mainly in patients undergoing dialysis, which itself represents a stress and decreased quality of life. However, experimental and causal studies are scarce. Our aim was to investigate dynamic changes in behavioral traits during the progression of CKD in an animal model. Thirty 12-week old male rats were used in this experiment. CKD was induced by a subtotal (5/6) nephrectomy. Two, 4, and 6 months after surgical induction of CKD, the open field, the light-dark box and the novel object recognition tests were conducted to assess the locomotor activity, anxiety-like behavior and the memory function of rats. Blood urea nitrogen (BUN), plasma concentration of creatinine (CREAT), albumin to creatinine ratio in urine (ACR) along with the renal histology were assessed to monitor the development and severity of CKD. In comparison to control rats, 5/6 nephrectomized rats had by 46–66% higher concentration of BUN during the whole follow-up period, as well as by 52% and by 167% higher CREAT and ACR, respectively, 6 months after surgery. Although the effect of time was observed in some behavioral parameters, nephrectomy did not significantly influence either locomotor activity, or anxiety-like behavior, or memory function of animals. Two and 4 months after surgery, animals moved shorter distance and spent less time in the center zone. However, the open-field ambulation returned back to the baseline level 6 months after CKD induction. Although nephrectomized rats displayed impaired kidney function as early as 2 months after surgery, no significant differences were found between the CKD and the control rats in any of the observed behaviors. Further studies are needed in order to evaluate whether behavioral abnormalities are related to severity of CKD or might be attributed to psychosocial aspect of end-stage renal disease and decreased quality of life in dialysis patients.

## Introduction

Chronic kidney disease (CKD) is defined as the glomerular filtration rate (GFR) lower than 60 ml/min per 1.73 m^2^ or a urinary albumin-to-creatinine ratio more than 30 mg/g. The severity of CKD can be divided into five stages based on GFR, fifth stage being the most severe and defined as a clearance creatinine <15 ml/min per 1.73 m^2^ (1). Proteinuria is another common clinical indicator of CKD and while it is part of the definition of CKD, it may not be present in the early stages of the disease. The last stages of CKD are also accompanied with poor prognosis and quality of life (2). Currently, the treatment options comprise either frequent dialysis or kidney transplantation (3), both of which further increase the healthcare costs as well as the incidence of secondary complications which arise either because of or along with CKD. It has been shown that CKD leads to cardiovascular disease (4), stroke (5) and decreased locomotor activity (6). Individual's quality of life can be further decreased by sexual dysfunction (7), neurological and mental disorders including anxiety and depression (7–11), as well as cognitive impairment (12–17) or epileptic seizures (18), all of which lead to increased socio-economic burden of affected people (19). Although, the exact pathophysiological mechanisms underlying the neurological complications of CKD remain unclear, there are several factors that might play a role, e.g., uremic encephalopathy (20), oxidative stress (21–23) and inflammation (21, 22).

Our previous research investigated the behavioral effects of long-term CKD in rats. Although no significant differences in motoric functions, depression-like behavior or memory impairment were found after 9 months (24), the study was limited to animals with CKD stage I-III. Animals with end-stage renal disease (CKD stage IV-V) were not included in behavioral testing due to high mortality rates in the first 9 months. Additionally, the animals were not tested throughout the stages of CKD development. Therefore, in present experiment, we aimed to examine changes in locomotion, memory and anxiety-like behavior 2, 4, and 6 months after CKD induction in a subtotal (5/6) nephrectomy rat model.

## Materials and Methods

### Animals

Twelve-week old male Wistar rats (*n* = 30) obtained from Anlab (Prague, Czech Republic) were used. The animals were housed in groups (*n* = 4/cage) and maintained in 12:12 h light/dark cycle under controlled condition (temperature 25 ± 2°C and humidity 55 ± 10%) with *ad libitum* access to food and water. The experiment was approved by the Ethics committee of the Institute of Pathological Physiology (Faculty of Medicine, Bratislava, Slovakia) and performed according to the Slovak legislation. During the experiment, two animals died in sham group (after 2 months) and three in 5/6 nephrectomized group (one after the surgery and two after 4 months).

### Surgery

The animals were randomly divided into subtotally nephrectomized (*n* = 17, 5/6nx) and sham-operated groups (*n* = 13, sham). Subtotal, 5/6 nephrectomy was performed in two steps with a 2-week reconvalescence between surgery steps: (1) the upper (1/3) and lower (1/3) poles of the left kidney were excised, (2) the whole right kidney (3/3) was excised (24). Both surgical procedures were carried out under general anesthesia by ketamine (100 mg/kg)/xylazine (10 mg/kg) administered by intraperitoneal injection. Incision was made on the left or right side of the abdomen respective to the side of surgery. The renal vessels were ligated and the kidney was decapsulated before excision. At the first surgical step, Gelaspon (Chauvin Ankerpharm GmbH, Rudolstadt, Germany) was used to stop the bleeding. The incisions were then sutured in two layers by absorbable Chirlac 4-0 silk (Chirmax, Modrany, Czech Republic) and non-absorbable Prolene 4-0 (Chirmax Modrany, Czech Republic) atraumatic sutures.

### Kidney Function

The 5/6 nephrectomy-induced CKD was confirmed by kidney function assessment. Before the surgery, as well as 2, 4, and 6 months after the surgery, the animals were placed into the metabolic cage for urine collection, and subsequently blood was taken from the tail vein. The body weight of animals was measured at every time point before placing the animals into the metabolic cage. Blood urea nitrogen (BUN) and plasma creatinine (CREAT) concentrations, as well as albumin to creatinine ratio in urine (ACR) were evaluated. Urea in plasma was measured by spectrophotometric assay using commercially available kits (Urea Nitrogen Colorimetric Detection Kit, Arbor Assays, Ann Arbor, USA). Creatinine in urine was assessed by Jaffe method, adding NaOH and picric acid (ratio 5:1) to the samples and measuring the absorbance at 492 nm (25). The concentration of urinary proteins was measured by the pyrogallol red-molybdate method, reading the absorbance at 592 nm (26).

### Histopathological Analysis

At sacrifice of the animals, the remnant or whole left kidney was dissected in 5/6nx and sham-operated rats, respectively, and fixed in 4% formaldehyde. Subsequently, the paraffin-embedded kidneys were cut in 3-μm sections and stained with Periodic acid Schiff (PAS) reagent. The incidence of focal glomerulosclerosis (*n* = 10 rats/group) was determined by a skilled examiner blinded to the groups, according to the procedure described previously (27).

### Behavioral Testing

#### Open Field Test

In the open field test (OF), the animals were tested for locomotor activity and anxiety-like behavior before the surgery (baseline), as well as 2, 4, and 6 months after the surgery. Each animal was individually placed into the PhenoTyper cage (Noldus Information Technology, Wageningen, Netherlands) and observed for 12 h during the dark part of the light/dark cycle. The square shaped arena (45 × 45 cm) was virtually divided into a center (20 × 20 cm) and a border zone. Locomotor activity and anxiety-like behavior were assessed, evaluating total distance moved during the observation period, and time spent in the center zone relative to the total time, respectively.

#### Light-Dark Box

Besides the open field test, animals were tested also in the light-dark box (LD) for anxiety. The apparatus (40 × 55 cm) consisted of an illuminated light chamber (250 lx) connected with a lid-covered dark chamber via passage, allowing free movement of the animals between the two chambers. The animals were placed into the light chamber and observed for 5 min. Percentage of the total time spent in the light chamber was assessed as an index of anti-anxiety behavior.

#### Novel Object Recognition

In addition to anxiety-like behavior, animals were also tested for recognition memory at 2, 4, and 6 months after the surgery. The novel object recognition test (NOR) was conducted in the familiar PhenoTyper cage. In the first round, the rats were exposed to two identical objects for 5 min, and time spent interacting with any of the objects was analyzed. One hour later, the animals were replaced into the arena and allowed to explore the arena for 5 min. In this round, one of the two objects was swapped for a novel object, which differed in shape, material and color from the familiar object. To eliminate preference of any of the objects due to its features, the objects were randomly selected as familiar or novel for each animal. In addition, to avoid side preference artifact, the position of the novel object in left or right side was systematically altered between the trials. According to the time interacting with the familiar and novel object, the preference for a novel object was calculated [interaction with novel object/(interaction with novel + familiar object)^*^100].

### Statistical Analysis

Data were statistically analyzed using GraphPad Prism version 6.00 for Windows (GraphPad Software, La Jolla, CA, USA). Two-way ANOVA was used (one factor: surgery, another factor: time) to evaluate the results. Bonferroni multiple comparison test was used to compare the groups in several time points, as well as to analyze the dynamical changes across the follow-up period in several groups. Due to the mortality during the experiment, the number of animals analyzed at several time points was altered and is reported in the charts. Statistical significance was considered when *p* < 0.05. Data are presented as mean plus standard deviation in the figures.

## Results

**Figure 1:**
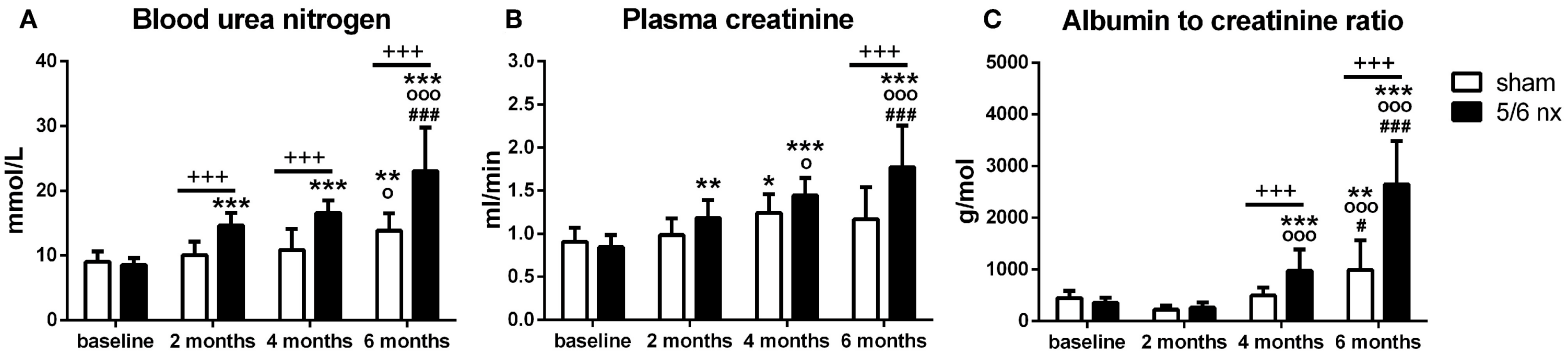
Kidney functions assessed by blood urea nitrogen **(A)**, creatinine in plasma **(B)** and albumin to creatinine ratio in urine **(C)**. Data are presented as mean + SD. ^+++^*p* < 0.001: between-group comparisons, ^*^p < 0.05, ^**^p < 0.01, and ^***^p < 0.001: comparisons to baseline, °*p* < 0.05 and °°°*p* < 0.001: comparisons to 2 months, ^#^*p* < 0.05, ^###^*p* < 0.001: comparisons to 4 months; sham group: *n* = 13 at 2 months, *n* = 11 at 4 and 6 months; 5/6nx groups: *n* = 16 at 2 and 4 months, *n* = 14 at 6 months; nx, nephrectomized.

Nephrectomy did not affect body weight of the animals (*F* = 0.01, *p* = 0.92), but a main effect of time (*F* = 343, *p* < 0.001) was observed (data not shown). In all of the assessed markers of renal failure (Figure 1), two-way ANOVA showed significant effect of time (BUN: *F* = 41.6, *p* < 0.001, Figure 1A; CREAT: *F* = 26.4, *p* < 0.001, Figure 1B; ACR: *F* = 84.8, *p* < 0.001, Figure 1C), surgery (BUN: *F* = 61.0, *p* < 0.001; CREAT: *F* = 20.9, *p* < 0.001; ACR: *F* = 47.8, *p* < 0.001), and interaction between time and surgery (BUN: *F* = 10.9, *p* < 0.001; CREAT: *F* = 6.61, *p* < 0.001; ACR: *F* = 26.8, *p* < 0.001).

The rats did not differ in BUN (*t* = 0.44, *p* > 0.99), CREAT (*t* = 0.63, *p* > 0.99) or ACR (*t* = 0.64, *p* > 0.99) at baseline. In 5/6nx rats, the concentrations of BUN, CREAT, and ACR have gradually increased over time. Two months after surgery, 5/6nx rats had by 46% (*t* = 3.83, *p* < 0.001) higher BUN concentration in comparison to control rats. Four months after surgery, BUN as well as ACR was by 53% (*t* = 4.82, p < 0.001) and by 97% higher (*t* = 3.12, *p* < 0.01), respectively, in 5/6nx rats than in sham-operated rats. Six months after surgery, 5/6nx rats have by 66% higher BUN (*t* = 6.93, *p* < 0.001), by 52% higher CREAT (*t* = 5.35, *p* < 0.001), and by 167% higher ACR (*t* = 10.5, *p* < 0.001) in comparison to control rats.

The increase in BUN (*t* = 5.45, *p* < 0.001) and CREAT (*t* = 3.62, *p* < 0.01) in 5/6nx rats have showed from 2 months, and in ACR (*t* = 4.52, *p* < 0.001) from 4 months after surgery when compared to the baseline. Six month after surgery, BUN was increased by 170% (*t* = 13.0, *p* < 0.001), CREAT by 110% (*t* = 9.61, *p* < 0.001), and ACR by 640% (*t* = 16.2, *p* < 0.001) in 5/6nx rats in comparison to the baseline. The levels of these parameters of kidney functions in 5/6nx rats at 6 months after surgery were increased also when compared to 2 month (BUN: *t* = 7.17, *p* < 0.001; CREAT: *t* = 6.11, *p* < 0.001; ACR: *t* = 16.6, *p* < 0.001) or to 4 months (BUN: *t* = 5.71, *p* < 0.001; CREAT: *t* = 3.38, *p* < 0.001; ACR: *t* = 11.7, *p* < 0.001), as well as at 4 months when compared to 2 months (CREAT: *t* = 2.83, *p* < 0.05; ACR: *t* = 5.14, *p* < 0.001). An increase in BUN concentration and ACR was found also in control rats when compared 6 months to baseline (BUN: *t* = 3.59, *p* < 0.01; ACR: *t* = 3.37, *p* < 0.01), to 2 months (BUN: *t* = 2.83, *p* < 0.05; ACR: *t* = 4.78, *p* < 0.001), or to 4 months (ACR: *t* = 3.00, *p* < 0.05). A slight increase in plasma CREAT was shown in control rats at 4 months after surgery, in comparison to baseline (*t* = 3.18, *p* < 0.05).

**Figure 2:**
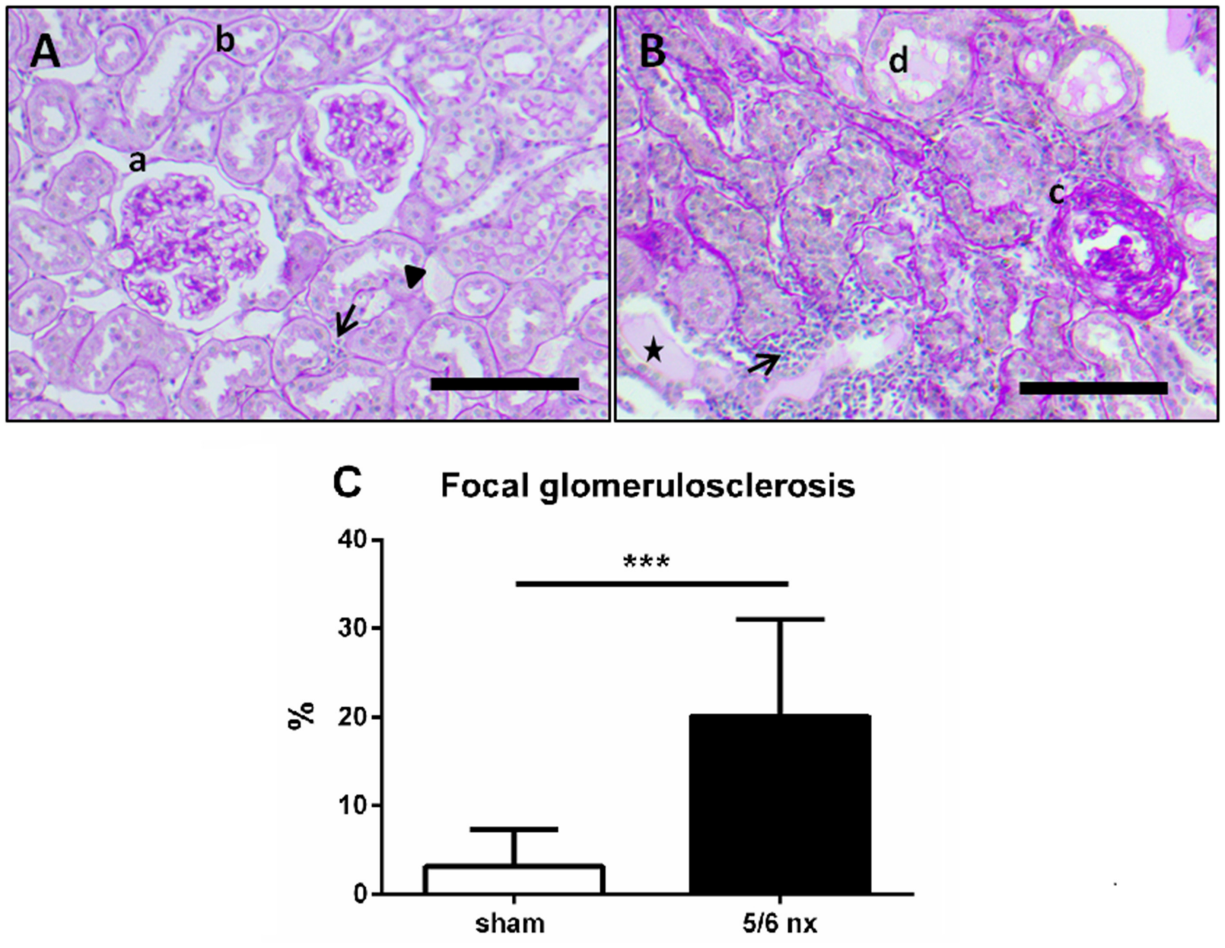
Representative pictures of renal histology in a sham-operated **(A)** and 5/6nx rat **(B)**, and the incidence of focal glomerulosclerosis **(C)**. In sham-operated rats **(A)**, only a mild age-related alteration was found in renal parenchyma. While glomeruli were not sclerotic (a) and the endothelial cells of tubuli (b) were normal, tickening of the tubular basement membrane (triangle), and a slight infiltration of lymphocytes (arrow) was shown. Kidney damage in 5/6nx rats **(B)** was confirmed by glomerulosclerosis (c), tubular dilation and atrophy (d), perivascular infiltration of lymphocytes (arrow), and proteinuric tubular casts (asterisk). Scale bars represent 100 μm (magnification 100×). The incidence of glomerulosclerosis was significantly higher in 5/6nx rats in comparison to control rats. Data are presented as mean + SD. ^***^p < 0.001, *n* = 10/group; nx, nephrectomized.

At sacrifice, only mild age-related alterations were found in renal architecture of sham-operated rats, such as thickening of tubular basement membrane and infiltration of lymphocytes, while glomeruli and tubuli were normal (Figure 2A: a–c), 5/6nx rats showed progressive renal injury, indicated by adhesion of the glomerulus to Bowman's capsule, thickening of Bowman's capsule and expansion of the collagen deposition in mesangium, proteinuric tubular casts, tubular dilation, and perivascular infiltration of lymphocytes (Figure 2B: d–f). Incidence of focal glomerulosclerosis was 6-fold higher in 5/6nx rats in comparison to control rats (*t* = 4.56, *p* < 0.001, Figure 2C).

**Figure 3:**
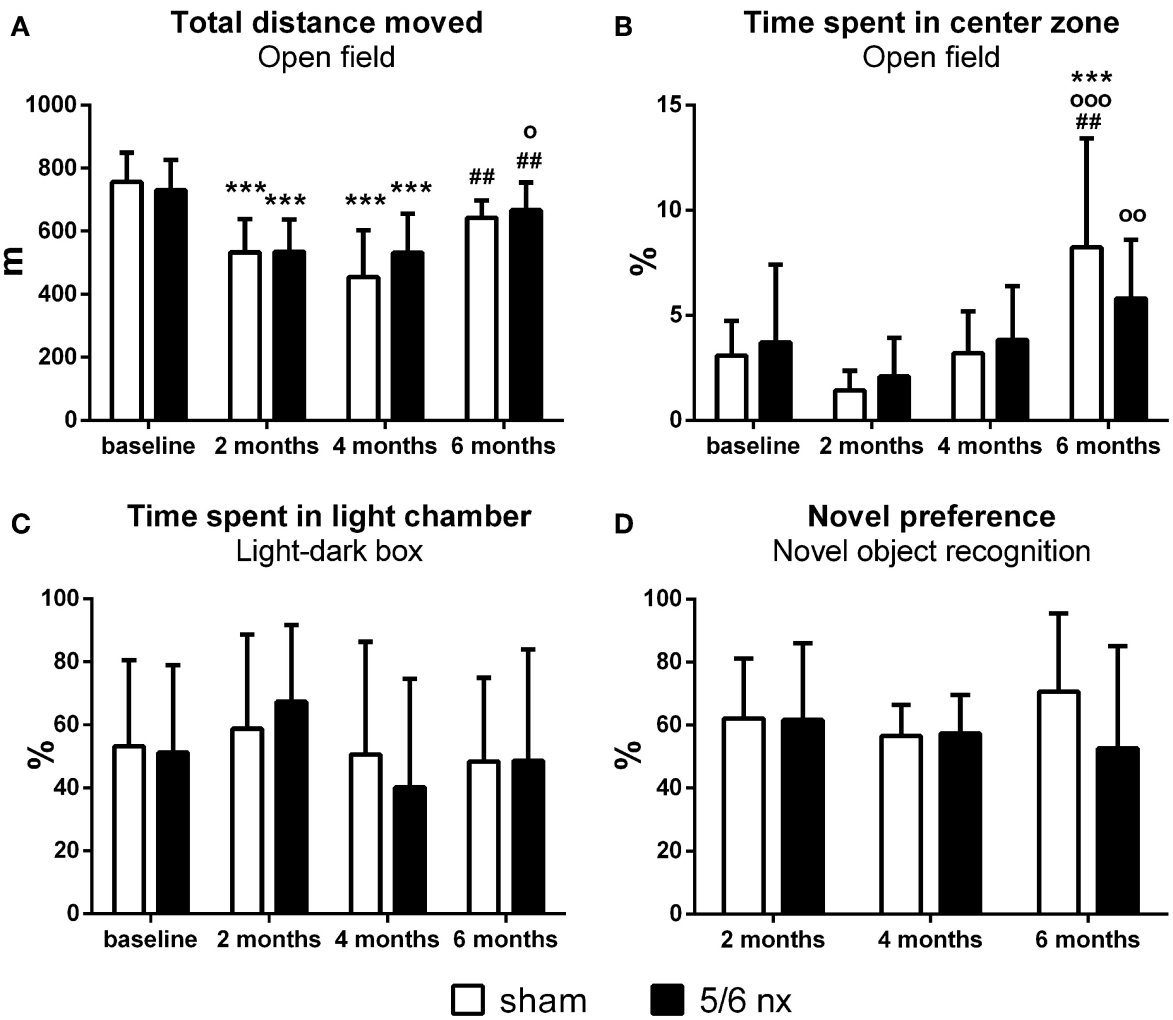
Locomotor activity, anxiety-like behavior and memory assessed by distance moved **(A)**, time spent in center zone of the open field **(B)** and in light chamber of the light/dark box **(C)** relative to the total time of testing, and interactions with novel object relative to the interactions with both, the familiar and novel objects **(D)**. Data are presented as mean + SD. ^***^p < 0.001: comparisons to baseline, °*p* < 0.05, °°*p* < 0.01, and °°°*p* < 0.001: comparisons to 2 months, ^##^*p* < 0.01: comparisons to 4 months; sham group: *n* = 13 at baseline and 2 months, *n* = 11 at 4 and 6 months; 5/6nx groups: *n* = 17 at baseline, *n* = 16 at 2 and 4 months, *n* = 14 at 6 months; nx, nephrectomized.

Prior to surgery, the animals did not differ in general locomotor activity (OF: *t* = 0.57, *p* > 0.99, Figure 3A), or anxiety-like behavior (OF: *t* = 0.53, *p* > 0.99, Figure 3B; LD: *t* = 0.18, *p* > 0.99, Figure 3C). No effect of surgery was found in assessed behavioral parameters (locomotor activity OF: *F* = 0.80, *p* = 0.37; anxiety-like behavior OF: *F* = 0.05, *p* = 0.82, LD: *F* = 0.02, *p* = 0.88; novel object preference: *F* = 1.12, *p* = 0.29, Figure 3D), at any of the time points. Two-way ANOVA revealed significant effect of time on the distance moved (*F* = 26.9, *p* < 0.001) and time spent in the center zone of the open field (*F* = 14.6, *p* < 0.001), but neither novel object preference (*F* = 0.32, *p* = 0.73) nor time spent in light chamber of LD (*F* = 1.56, *p* = 0.21) was influenced by time.

Regardless of the nephrectomy, the rats displayed lower locomotor activity at 2 months (sham: *t* = 6.06, *p* < 0.001; 5/6nx: *t* = 4.87, *p* < 0.001), as well as at 4 months after surgery (sham: *t* = 6.25, *p* < 0.001; 5/6nx: *t* = 4.88, *p* < 0.001) in comparison to the baseline. Additionally, a slight (statistically non-significant) decrease in center-time was observed in both groups at 2 months. Six months after CKD induction or sham operation, the locomotor activity of the animals was again similar to the baseline, thus higher than at 2 months (5/6nx: *t* = 3.21, *p* < 0.05) and 4 months (sham: *t* = 3.81, *p* < 0.01; 5/6nx: *t* = 3.25, *p* < 0.01). At this time, rats in both group spent significantly more time in the center zone when compared to the previous time points (sham: *t* = 4.08, *p* < 0.001 vs. baseline; *t* = 5.72, *p* < 0.001 vs. 2 months; *t* = 3.77, *p* < 0.01 vs. 4 months; 5/6nx: *t* = 3.46, *p* < 0.01 vs. 2 months). Sham as well as 5/6nx rats showed a decreasing tendency to explore the objects in the novel object recognition test. At four (sham: *t* = 3.04, *p* < 0.01) and 6 months after surgical intervention (sham: *t* = 2.69, *p* < 0.05; 5/6nx: *t* = 4.23, *p* < 0.001) the total interaction time was decreased almost by 50% than at 2 months.

## Discussion

In our experiment, decreased renal function in 5/6nx rat model of CKD was indicated by increased concentration of BUN, as soon as at 2 month after surgery. Furthermore, 6 months after CKD induction, renal histopathological alterations, as well as increased BUN, CREAT, and ACR clearly indicated renal failure in 5/6nx rats. However, during the 6 months follow-up period, we did not find any effect of CKD induction on locomotor activity, anxiety-like behavior or memory function of rats. Regardless of nephrectomy, locomotor activity and anxiety-like behavior of rats were characterized by high variability and fluctuation over time, particularly in the open field test. Our results contradict to the clinical studies suggesting an association between renal failure and behavioral abnormalities.

Chronic kidney disease is frequently associated with neurological disorders affecting both peripheral and central nervous system resulting in behavioral abnormalities, including mental and cognitive dysfunctions (28–30). It has been shown that mild CKD may lead to activation of limbic system, central sympathetic, stress- and pain-related brain areas (31). Some CKD-related neurological complications have been attributed to impaired permeability of the blood-brain barrier (22, 32, 33), neuroinflammation, and oxidative stress (21–23). Neurotoxicity induced by numerous substances, such as urea, accumulated in blood during kidney disease (34–36) might also play a role (33).

Numerous clinical studies have indicated an association between physical activity and cardiovascular (37) and metabolic co-morbidities (38) in CKD patients. Additionally, some animal models of moderate and severe renal insufficiency showed that CKD may lead to reduced locomotor and exploratory activity of rats (36, 39, 40). In line with this, we expected a decline in locomotor activity of CKD rats concurrently with deterioration of kidney functions. However, both 5/6nx rats and sham animals displayed decrement in ambulatory activity 2 and 4 months, followed by increment 6 months after CKD induction. This indicates that fluctuating mobility of CKD rats is not related to neurological symptoms caused by kidney damage, but rather results from other causes, e.g., repeated testing of the animals (41).

Reduced quality of life of patients with CKD is associated with high prevalence of anxiety and depressive disorders (7–10). However, animal experiments have brought contradictory results. Although depressive-like behavior was found in some animal models of CKD (21, 32), it has been shown several times that animals with CKD exhibit decreased emotionality (39) and low-level anxiety (24, 32, 42). The anxiolytic effect of CKD was indicated mainly by reduced time spent in the dark chamber of light/dark box, while it was not manifested in other tests assessing anxiety, such as elevated plus maze or open field test (24, 32, 42). On the other hand, Kielstein et al. (36) found increased anxiety-like behavior in 5/6nx rats when tested on hole board. These results suggest a test-specific effect of CKD on anxiety. In our experiment, light/dark box and open field test were used to assess anxiety-like behavior and found no difference between CKD and control rats in any of the tests. The reason remains unclear, but different stages of kidney damage, due to different way of CKD induction, e.g., surgical (24, 31, 42, 43) vs. chemical (32, 36, 40, 44), should be considered. While in our experiment, 5/6nx rats did not differ from control rats in body weight, CKD was accompanied by reduced body weight in other rodent models (40), which might influence the performance of the animals in the behavioral tests. As previously shown, in our laboratory 5/6nx rats did not display any signs of behavioral abnormalities, while their glomerular filtration rate was lowered by 79% in comparison to controls. However, after 9 months, increased proteinuria, as well as increased concentration of plasma creatinine and urea was accompanied by decreased anxiety-like behavior (24). This indicates that in present experiment behavioral abnormalities could not manifest due to the shorter follow-up period. It should be noted that to examine the dynamics of behavioral changes from early to advanced stage of CKD in rats is challenging, particularly because of the high mortality of 5/6nx rats around 6–7 months after CKD induction (24).

Cognitive decline is one of the most common neurological symptoms in CKD patients (12–15, 17, 30). Numerous experiments have confirmed deficit in spatial- (23, 45), recognition- (21, 42, 45–47), as well as aversive-related memory (46–48) in CKD animals. On the contrary, there are also some experiments which failed to show memory-impairing effect of CKD (24, 49). Discrepancies among the results may arise from the variability of susceptibility to cognitive impairment in CKD animals (47), which may result from differences in underlying neurological condition. It has been suggested that in an animal model of Alzheimer's disease, CKD as co-morbidity may induce cognitive impairment (49). Additionally, age (50) as well as sex (51) may affect the prognosis and neurological effects of CKD.

To the best of our knowledge, present study is the first to examine locomotor activity, anxiety-like behavior and recognition memory at three different time points during CKD development in rats. Nevertheless, our experiment has some limitations. The major limitation is the repeated testing of the animals done in order to investigate potential alterations in behavior related to progression of renal failure, and to minimize the number of animals per group. In our study, plasma concentration of urea, creatinine and ACR were used to assess severity of kidney damage in several phases of CKD development. While higher concentration of urea was detected as early as 2 months after nephrectomy, ACR and plasma creatinine were higher only at 6 months after CKD induction when compared to control rats. Unfortunately, there is a lack of well-defined and unified scoring system to determine distinct CKD stages in rodents based on any of the markers of renal failure (e.g., BUN, creatinine clearance, proteinuria). Additionally, in numerous previous studies, the confirmation of CKD-induced impaired kidney function was missing (42) making comparison with published experiments on behavioral outcomes of CKD severity very difficult. Furthermore, there are several animal models of CKD differing in signs and symptoms (52, 53). Thus, the development of neurobehavioral disorders related to CKD may depend on the model used, which should be considered in future studies. Although animal models mimicking CKD offer relevant tools to examine causality behind several associations, it should be noted that in humans, the main causes of CKD are diabetes and hypertension (54). Therefore, the translational value and relevance of surgically induced CKD might be disputable in medicine. Furthermore, it should be considered that several behavioral and mental disorders in CKD patients may arise from primary disease (55–58) or from co-morbidities (11), as well as from psychosocial aspects of chronic illness (8, 19, 59).

In conclusion, 5/6 nephrectomy-induced renal failure was evident as early as 2 months after surgery, however, it did not lead to any behavioral changes in adult male rats. Furthermore, worsening renal functions were not related to decline in locomotor activity, cognition or emotion. The causes of behavioral abnormalities observed in CKD patients remain to be elucidated in further experiments, with particular attention to numerous co-factors influencing behavior, such as chronic stress, pain or social isolation.

## Data Availability Statement

All datasets generated for this study are included in the article/supplementary material.

## Ethics Statement

The animal study was reviewed and approved by Ethics committee of the Institute of Pathological Physiology (Faculty of Medicine, Bratislava, Slovakia).

## Author Contributions

ER performed nephrectomy and behavioral tests, analyzed data and prepared the figures, and drafted the manuscript. MM performed behavioral tests and drafted the manuscript. AG performed nephrectomy and measured biomarkers of kidney injury. DV-Y performed histological analysis of the kidneys. L'T designed the experiment, corrected the draft, and provided financial support. JH designed the experiment, analyzed data, and corrected the draft. All authors approved the final version of the manuscript.

### Conflict of Interest

The authors declare that the research was conducted in the absence of any commercial or financial relationships that could be construed as a potential conflict of interest.
